# Undeveloped Testes from Tobacco-Chewing

**Published:** 1882-12

**Authors:** 


### TOBACCO-CHEWING BOYS TAKE WARNING.—UNDEVELOPED TESTES ASSOCIATED WITH EARLY TOBACCO-CHEWING.

Mr. R. Clement Lucas, Senior Assistant Surgeon to Guy's Hospital, reports a case in the *British Medical Journal* of this sad condition which he confidently attributes to tobacco chewing. His condition is thus delineated: "His penis was small, and the prepuce rather long. The testicles were remarkably small, neither being larger than a French bean, or, perhaps, what more nearly expresses their size and shape, no larger than the testes of a rabbit.—*Louisville Med. News.*

